# Bioaccessible Sulforaphane Drives Macrophage Migration and Differentiation by Reprogramming the Interleukin Profile in Intestinal Inflammation

**DOI:** 10.1002/biof.70101

**Published:** 2026-04-13

**Authors:** Sonia Medina, Concepción Medrano‐Padial, Cristina García‐Viguera, Raúl Domínguez‐Perles

**Affiliations:** ^1^ Laboratorio de Fitoquímica y Alimentos Saludables (LabFAS), CSIC, CEBAS Campus Universitario de Espinardo Murcia Spain

**Keywords:** bioaccessibility, broccoli by‐products, interleukin modulation, intestinal inflammation, macrophage polarization, sulforaphane

## Abstract

Inflammatory bowel disease (IBD) arises from dysregulated interactions between the immune system and the intestinal microenvironment. Finding new therapeutic targets could help to develop treatments that attenuate its severity. The present study investigated the immunomodulatory potential of bioaccessible sulforaphane (SFN, 0.100 μg/mL) from broccoli by‐products. Interestingly, the main results evidenced that at this physiological concentration, SFN contributed to reducing the secretion of pro‐inflammatory interleukins (IL‐1*β*, IL‐6, IL‐17, IL‐18, IL‐23, TNF‐*α*) by intestinal epithelium up to ~56%, whereas enhancing anti‐inflammatory cytokines (IL‐10, IL‐4, IL‐13) between ~24% and ~71%. These changes adjusted the proportion of CD86^+^ and CD206^+^ during macrophage differentiation, associated with the prevention of immune‐mediated IBD. In addition, a reduction in the expression of macrophage‐dependent pro‐inflammatory cytokines and an augmentation of the tolerogenic classes were observed. The combined use of intestinal epithelial (Caco‐2) and monocytic (THP‐1) cell lines established an in vitro model of the epithelium‐macrophage crosstalk, thereby enhancing the physiological relevance of our findings. These results were confirmed using a pure SFN‐based model system, which demonstrated SFN's contribution to the anti‐inflammatory properties of broccoli stalk and bridged the gap between in vitro findings and potential dietary/therapeutic applications. Thereby, this work demonstrated that dietary SFN contributes to a large extent to the reshaping capacity of the phytochemical burden of broccoli stalks, on the interleukin profile secreted by epithelium and macrophages, as well as the macrophage differentiation, thus supporting the valorisation of broccoli by‐products for preventing and managing inflammatory conditions, such as IBD.

## Graphical abstract

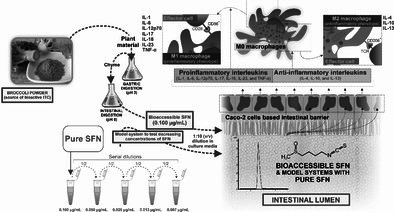

**Abbreviations**
ANOVA: analysis of variance
BSA: bovine serum albumin
Caco‐2: human colon adenocarcinoma cell line
COX‐2: cyclooxygenase‐2
DIM: 3,4‐diindolylmethane
DSS: dextran‐sulfate sodium
E: erucin
ELISA: enzyme‐linked immunosorbent assay
EMEM: eagle's minimum essential medium
FBS: fetal bovine serum
GB: glucobrassicin
GE: glucoerucin
GI: glucoiberin
GR: glucoraphanin
GSL: glucosinolate
HGB: hydroxy‐glucobrassicin
I3C: indole‐3‐carbinol
IB: iberin
IBD: intestinal bowel disease
IBD: intestinal bowel disease
IFN‐*γ*
: interferon‐gamma
IL: interleukin
ITC: isothiocyanates
LC–MS: liquid chromatography‐mass spectrometry
LOD: limit of detection
MGB: methoxy‐glucobrassicin
MIF: macrophage migration inhibitory factor
MPO: myeloperoxidase
MRM: multiple reaction monitoring
NGB: neo‐glucobrassicin
PBS: phosphate‐buffered saline
PMA: phorbol 12‐myristate 13‐acetate
PVDF: polyvinylidene fluoride
RPMI: roswell park memorial institute medium
SD: standard deviation
SFN: sulforaphane
THP‐1: human monocytic cell line
TNF‐*α*
: tumor necrosis factor alpha

## 1 Introduction

The prevalence of inflammatory bowel disease (IBD) is projected to affect 1% of the population by 2030 [1, 2]. This involves a group of diseases (e.g., Crohn's disease and ulcerative colitis) that arise from dysregulated interactions among the immune system, gut microbiota, and environmental factors [3], impairing quality of life [4]. Interestingly, the dietary patterns determine the gut microbiota profile, contributing to the initiation of autoreactive immune responses [5], whereas, alternatively, certain dietary molecules may attenuate IBD symptoms [6], motivating the search for compounds involved in these pathogenic mechanisms.

Among vegetable foods, broccoli ( *Brassica oleracea var. italica* ) stands out for its content of organosulfur compounds [7]. This class of phytochemicals include glucosinolates (GSL), which feature a *β*‐D‐thioglucose linked to a sulfonated oxime moiety and a variable side chain (R) [8]. The biological activity of these compounds arises from their hydrolysis products (e.g., isothiocyanates (ITC) or nitriles), which are generated enzymatically by plant *β*‐thioglucosidase (myrosinase) [9] or during gastrointestinal digestion [10], and can interact with inflammation‐related molecules to exert preventive or therapeutic effects [11].

To date, the anti‐inflammatory activity of SFN has been demonstrated in vivo, mainly by monitoring changes in interleukin profiles in models of dextran sulfate sodium (DSS)‐induced intestinal inflammation [12, 13, 14, 15]. These models have also provided evidence on SFN's ability to prevent colonic shortening, histological damage, myeloperoxidase (MPO) activity, and gut microbiota dysbiosis. Alternatively, in vitro available mechanistic evidence indicates that SFN can attenuate inflammatory signaling in the intestinal epithelium by reducing the expression of COX‐2 and derived oxylipins, as well as the secretion of pro‐inflammatory interleukins [11, 16], thus promoting a balanced M1/M2 phenotype and limiting epithelial damage under inflammatory conditions [17]. Nevertheless, to some extent, one relevant weakness of this strategy is the evaluation of isolated SFN at supraphysiological concentrations. As a consequence, it remains unclear whether epithelial immunomodulation induced by bioaccessible SFN (part of a complex mixture released during digestion from plant‐based food [10], where SFN represents a major but not exclusive bioactive constituent and where it is characterized by poor stability) is sufficient to drive coordinated macrophage recruitment and polarization. Therefore, to date, it is urgent to demonstrate the capacity of bioaccessible SFN to modulate the epithelial response to inflammation and macrophage recruitment/activation. The advance from the current knowledge could be achieved through in vitro interventions, thus bridging in vitro efficacy with dietary relevance [18].

From this point, the present work hypothesizes that such modifications in local tissues may influence adaptive immune responses, such as the migration and differentiation of macrophages into M1‐CD86^+^ (pro‐inflammatory) or M2‐CD206^+^ (anti‐inflammatory) cells [3, 19], key determinants of IBD progression [19], which should be explored in vitro [20, 21, 22, 23]. This knowledge is needed because these phenotypes have opposite effects on the immune response, either promoting or suppressing inflammation [24]. Therefore, evaluating SFN at the attainable concentrations after gastrointestinal digestion is essential to identify the factors that contribute to the restoration of immune homeostasis and the mitigation of IBD symptoms [6, 25]. In this frame, model systems with individual compounds at equivalent concentrations would allow dissecting the contribution of specific molecules [26].

Building on previous evidence, the present study aims to advance this knowledge by linking epithelial immunomodulation to downstream functional immune responses concerning macrophage recruitment and polarization, key events in the initiation and resolution of intestinal inflammation. Furthermore, by combining the assessment of bioaccessible fractions with a model system based on graded concentrations of pure SFN, it was possible to estimate the contribution of SFN within a complex bioactive matrix.

## 2 Materials and Methods

### 2.1 Chemicals and Reagents

The standards of glucoraphanin, glucoerucin, glucoiberin, glucobrassicin, hydroxyl‐glucobrassicin, methoxyglucobrassicin, neoglubobrassicin, sulforaphane, erucin, indole‐3‐carbinol, 3,4‐diindolylmethane, and iberin (GR, GE, GI, GB, HO‐GB, MeOH‐GB, NeoGB, SFN, ER, I3C, DIM, and IB, respectively) were purchased from Phytoplan GmbH (Heidelberg, Germany). Trypsin‐ethylenediaminetetraacetic acid (EDTA), Eagle's Minimum Essential Medium (EMEM), L‐glutamine, foetal bovine serum (FBS), penicillin/streptomycin, and essential amino acids were sourced from Gibco (ThermoFisher Scientific, Madrid, Spain). Flat‐bottom 24‐well plates and polycarbonate membrane inserts (5 μm pore size) were obtained from Corning (New York, NY, USA). Phorbol 12‐myristate 13‐acetate (PMA), IL‐1β, IL‐4, and IL‐13 were sourced from R&D Systems (Minneapolis, MN, USA). CoraLite Plus 647 Anti‐Human CD86 and CD206 antibodies were obtained from Proteintech (ThermoFisher Scientific, Madrid, Spain). All LC–MS solvents were obtained from J.T. Baker (Phillipsburg, NJ, USA). Ultrapure water was produced using a Millipore water purification system (Bedford, MA, USA). For quantifying the various human interleukins monitored (IL‐1, IL‐4, IL‐6, IL‐10, tumor necrosis factor alpha (TNF‐*α*), IL‐12p70, IL‐13, IL‐17, and IL‐23). Enzyme‐linked immunosorbent assay (ELISA) kits were purchased from Invitrogen‐Thermofisher Scientific (Madrid, Spain). Additionally, the human IL‐13 ELISA kit was provided by Proteintech (Munich, Germany).

### 2.2 Plant Material

Commercial seeds of broccoli plants ( *B. oleracea* Lam. italica, cv. ‘Parthenon’) were obtained from Cytoplasmic Male Sterility provided by SAKATA Seed Ibérica (Alicante, Spain). Broccoli plants were cultivated in the fall–winter cycle of 2024 in the Experimental farm of CEBAS‐CSIC, “La Matanza” (Santomera, Murcia, SE Spain; 38°6′14″ N, 1°1′59″ W). Harvesting was performed when plants presented mature commercial flowering heads with quality parameters corresponding to the “marketable” class, and inflorescences were manually separated from the stalks. The period between sampling and processing was less than 4 h to avoid degradation. Once in the lab, broccoli stalks were processed according to the conditions described by Costa‐Pérez et al. to obtain a stabilized material providing high concentrations of bioaccessible SFN [10]. The dehydrated samples were ground into a fine powder, stored, and protected from light until GSL and ITC extraction, following the methodology described in the literature [27]. Alternatively, this material underwent an in vitro gastrointestinal digestion to evaluate the SFN bioaccessibility.

### 2.3 In Vitro Simulated Gastrointestinal Digestion

Gastrointestinal digestions were performed on dehydrated broccoli stalk powder following the previously published methodologies [28, 29], with minor modifications [27, 30]. Briefly, the digestion's simulation was carried out using pepsin, pancreatin, and pancreatic lipase as digestive enzymes. After digestion, the bioaccessible fractions were filtered through a 0.22 μm polyvinylidene fluoride (PVDF) membrane (Millipore, MA, USA) and stored at −80°C until analysis by liquid chromatography coupled to mass spectrometry (LC–MS).

### 2.4 HPLC‐PDA‐ESI‐MSn‐Based Qualitative and Quantitative Profiling of Glucosinolates

The chromatographic separation and spectrometry analysis of the individual GSLs in both the analytical extracts and gastrointestinal digestion products was performed using the methodology already described in the literature [11]. Target analytes were identified and quantified from chromatograms recorded at 227 nm, using freshly prepared calibration curves, and the concentration was expressed as milligrams per kilogram of dry weight (mg/kg dw).

### 2.5 Assessment of the Bioaccessible Fraction of Broccoli Stalks for Sulforaphane Content by UHPLC‐QqQ‐MS/MS Analysis

The concentration of SFN in the broccoli stalk's bioaccessible fraction was obtained based on the retention time, parental mass, and specific fragmentation pattern, which were monitored through quantification and confirmation transitions using Multiple Reaction Monitoring (MRM) by UHPLC‐QqQ‐MS/MS analysis [31, 32]. The concentration of SFN was calculated using standard curves freshly prepared with authentic standard on each analysis day and expressed as milligrams per kilogram dry weight (mg/kg dw) and its equivalent in μg/mL for comparative purposes.

### 2.6 Cell Lines, Culture, and Experimental Conditions

The human colon adenocarcinoma (Caco‐2, HTB‐37) and human monocytic (THP‐1, TIB‐202) cell lines were sourced from the American Type Culture Collection (ATCC, Rockville, MD, USA) and maintained at passage numbers between 20 and 30 for the experiments. Caco‐2 cells were grown using the culture medium and methodology described to obtain a monolayer model of the intestinal barrier [11], which represents, in vitro, the ultrastructure and functionality of human intestinal epithelium. Briefly, Caco‐2 cells were grown in EMEM, supplemented with 2 mM L‐glutamine, 10% fetal bovine serum (FBS), 1% penicillin/streptomycin (5000 U/mL), and 1% non‐essential amino acids at 37°C in a humidified atmosphere containing 5% CO_2_. To obtain a Caco‐2‐based model of intestinal barrier, cells were seeded at a density of 1.5 × 10^5^ in a 24‐well plate and once confluent, they were allowed to differentiate into an in vitro cell system mimicking the absorptive and barrier functions of human intestinal epithelium for 21 days [33], replacing culture media every 48–72 h. Once differentiated, enterocyte‐like Caco‐2 cells were used to evaluate their quantitative response (both pro‐ and anti‐interleukin profiles) and macrophage chemotactic capacity under inflammatory conditions, as well as their modulation by bioactive SFN.

Moreover, THP‐1 cells were grown using the methodology described by Domínguez‐Perles et al. [6], with minor modifications. In short, despite the growth media for THP‐1 cells being RPMI 1640 supplemented with 10% FBS, penicillin/streptomycin (5000 U/mL), and 2 mM L‐glutamine (final concentration), to overcome side effects due to the stress caused by exposing cells to different growing conditions during the experiments, they were adapted to grow in the Caco‐2 growth media (EMEM medium supplemented with 2 mM L‐glutamine, 10% foetal bovine serum (FBS), 1% penicillin/streptomycin (5000 U/mL), and 1% non‐essential amino acids). The growing conditions and experimental settings for THP‐1 cells concerning migration and differentiation experiments are described in subsequent subsections.

### 2.7 Macrophage Migration Assay

To retrieve evidence of the capacity of the bioaccessible fraction of broccoli stalks, Caco‐2 cells were differentiated into ciliated cells following the methodology described in Subsection 2.6 (Cell Lines, Culture, and Experimental Conditions) [34]. Once obtained functional epithelial cells, the culture medium was replaced by fresh growth media supplemented with the bioaccessible fraction (90:10, v/v) containing 0.0100 μg/mL SFN (final concentration in the well) (or serial dilutions of pure SFN starting at the bioaccessible concentration providing 0.0100, 0.0050, 0.0025, 0.0013, and 0.0007 μg/mL, final concentrations in the well) for 1 h, followed by treating cells with 25 ng/mL IL‐1β for 10 h, when the highest concentration of inflammation‐related interleukins is present in the growth media [35]. Untreated Caco‐2 cells and replicates exposed only to 25 ng/mL IL‐1β were considered negative and positive controls, respectively.

The migration of THP‐1 cells in response to chemotactic signals produced by Caco‐2 cells under inflammatory conditions was assessed using a 24‐well Transwell system with a pore diameter of 5 μm by applying the methodology described in the literature [6, 34]. Briefly, THP‐1 cells (1.0 × 10^5^ cells in 0.100 mL of serum‐free DMEM culture media supplemented with 2 mM L‐glutamine) were deposited into a 6.5 mm Transwell insert (apical compartment). This cell density and volume were selected based on previously reported optimisation studies to ensure adequate diffusion gradients while preventing pore saturation or cell overcrowding. THP‐1 monocytes were serum‐starved overnight before migration, and assays were performed in serum‐free medium. Inserts were placed over wells containing 500 μL of Caco‐2 cells' supernatants generated under the experimental conditions described above. Plates were incubated at 37°C for 24 h, a time point selected to allow sufficient migration while preserving cell viability and gradient stability. Migrated cells in the lower wells were quantified and expressed as a percentage of total seeded cells. All the conditions were performed in triplicate (*n* = 3).

### 2.8 Evaluation of the Ability of Sulforaphane to Modulate THP‐1 Monocytic Cell Differentiation Towards M1 and M2 Phenotypes

THP‐1 monocytes were seeded into 12‐well plates at a concentration of 10^6^ cells/mL and stimulated with 150 nM (~90 ng/mL) PMA for 24 h to differentiate monocytes into M0 macrophages according to previous optimisations of the differentiation process, based on cell morphology, adherence, and cell density, monitored by light microscopy. Following PMA stimulation, cells were washed twice without an additional resting period before exposure to polarizing stimuli or Caco‐2 supernatants. As an additional internal control and to confirm the capacity of M0 cells to differentiate into M1 and M2 (pro‐ and anti‐inflammatory macrophages), PMA‐treated THP‐1 cells were exposed to specific M1 and M2 polarizing stimuli (IL‐and IFNγ (20 ng/mL) / LPS (10 pg/mL) and IL‐4 (20 ng/mL)/IL‐13 (20 ng/mL) for 24 and 72 h, respectively [36]) (data not shown). Cell morphology and adherence were monitored by light microscopy. Within the experimental design, M0 macrophages were exposed to Caco‐2 cells' supernatants (produced according to the methodology described in Subsection 2.7) for 24 and 72 h to evaluate the capacity of the interleukin profile secreted into the culture media to induce macrophage polarization towards M1 (proinflammatory) or M2 (anti‐inflammatory) phenotypes, respectively.

The differentiation of macrophages and the frequency of the M1 and M2 phenotypes were determined by two‐color flow cytometry. After exposure to the differentiation stimuli, cells were washed with cold PBS and detached with EDTA 5 mM to minimize membrane receptor alteration. Cells were then harvested, washed with PBS, and processed for antibody staining. With this goal, the pellets obtained were incubated for 30 min with mouse anti‐human Coralite Plus‐647 fluorochrome‐labeled antibodies CD86 (for M1 pro‐inflammatory macrophages) and CD206 (for M2 anti‐inflammatory macrophages) at the concentrations recommended by the manufacturer (5 μL per 10^6^ cells in 100 μL of PBS). After incubation, the cells were washed with 1 mL PBS and centrifuged at 500 × g for 5 min. The supernatants were discarded, and cells were resuspended in PBS with 0.1% sodium azide and 0.5% bovine serum albumin (BSA). Flow cytometry was performed with a FACScalibur cytometer (Becton Dickinson, San Diego, CA, USA), and data were analyzed with FlowJo v10.10 software (Becton Dickinson, San Diego, CA, USA). A total of 5000 events per sample were collected, and the Mean Fluorescence Intensity (MFI) was determined for each marker.

### 2.9 Profiling Interleukin Environments

The quantitative interleukin profile, including pro‐inflammatory (IL‐1, IL‐6, IL‐12p70, IL‐17, IL‐18, IL‐23, and TNF‐*α*) and anti‐inflammatory (IL‐4, IL‐10, and IL‐13) classes, was assessed in the supernatant obtained with the experimental conditions detailed in Sections 2.7 and 2.8 using ELISA kits following the manufacturer's instructions.

### 2.10 Statistical Analysis

All experimental conditions were performed in triplicate (*n* = 3), and the data were expressed as the mean ± standard deviation (SD). According to the normal distribution and homogeneity of variance of the data (determined by the Shapiro–Wilk (< 50 samples) and Levene tests, respectively), the obtained results were subjected to one‐way analyses of variance (ANOVA). When statistical differences were identified, the variables were compared using Tukey's multiple range test. Significant differences were set at *p* < 0.05. All statistical analyses were performed with SPSS, version 29.0 (SPSS Inc., Chicago, IL, USA).

## 3 Results and Discussion

### 3.1 Organosulfur Compounds of Broccoli Stalks: Plant Material Versus Bioaccessible Fraction

To date, there is broad evidence supporting the interest of broccoli as a source of bioactive organosulfur compounds, which have been associated with the prevention of inflammation through their interaction with molecular mediators [11]. These compounds, particularly GSL and ITC, have been shown to modulate the secretion of both pro‐ and anti‐inflammatory interleukins by the intestinal epithelium and, subsequently, by recruited immune cells such as macrophages. As a result, GSL and ITC have been associated with a strong anti‐inflammatory potential [11].

Building on our previous work, which established the capacity of broccoli stalk‐derived bioaccessible fraction to attenuate inflammatory signaling at the epithelial level [11], the present study provides robust evidence on the immunological relevance of this modulation by focusing on an expanded interleukin network and its functional consequences on macrophage behavior. In this regard, for these bioactivities to be physiologically relevant, effective concentrations of the bioactive forms of organosulfur compounds (e.g., ITC) must be achieved at the intestinal level. In this context, previous work by Costa et al. described a specific dehydratation process at low temperature that maximizes SFN bioaccessibility from broccoli stalks [10, 11, 16], thereby providing a suitable framework to explore the downstream immunomodulatory effects.

Applying these processing conditions in the present study resulted in broccoli stalk material characterized by the following GSL profile: GR (2423.2 mg/kg dw) > GI and MGB (458.9 mg/kg dw, on average) > GE (235.8 mg/kg dw) > GB, HGB, and NGB (106.1 mg/kg dw, on average). The quantitative values recorded in the present work are consistent with the well‐established quantitative profile of broccoli [37]. This GSL profile allows envisaging the preponderant formation of the best‐characterized ITC, SFN, also boosted by the high concentration of GE, which is hydrolysed to form erucin, which is further converted into SFN [38]. This ITC profile would contribute extensively to the anti‐inflammatory power of the broccoli stalk‐based materials. However, the formation of SFN during gastrointestinal digestion and its efficient bioactivity need to be further confirmed.

Accordingly, in the bioaccessible fraction obtained after simulated gastrointestinal digestion of broccoli stalks performed in this study (mimicking the physiological process [28, 29]), no intact GSL were found above the limit of detection (> LOD). This observation likely reflects their rapid hydrolysis into ITC and nitriles under digestive conditions, in agreement with previous reports describing the instability of GSL during digestion and the consequent release of ITC and indoles into the intestinal lumen [10, 11].

Quantification of ITC in the bioaccessible fraction revealed that the digestion process released SFN at a concentration of 5.92 μg/g dw, corresponding to 0.099 μg/mL. This SFN level results from the hydrolysis of GR and GE into SFN and E, respectively, and the subsequent conversion of E into SFN during gastrointestinal digestion [10, 27, 31]. To date, this range of concentrations has not been tested for the anti‐inflammatory activity of SFN, which has typically been investigated at supraphysiological levels targeting specific molecular pathways associated with the pathogenesis of IBD [17, 39].

In this regard, the present work aims at establishing a direct link between experimentally generated bioaccessible SFN concentrations and the modulation of the intestinal epithelium's interleukin profile, thus contributing to the prevention of autoreactive immunological mechanisms associated with intestinal inflammation, such as macrophage recruitment and polarization (M1/M2) and cytokine‐mediated responses. Additionally, to further confirm the specific contribution of SFN, the anti‐inflammatory response observed for the bioaccessible fraction was compared with that elicited by matching concentrations of pure SFN (0.100 μg/mL) and serial dilutions up to 0.007 μg/mL (0.0100–0.0007 μg/mL, final concentration in wells after diluting 1:10 (v/v) in culture media to develop the functional assays).

### 3.2 Modulation of the Intestinal Epithelium Interleukin Profile by Bioaccessible Sulforaphane Under Inflammatory Conditions

As mentioned earlier, IBD is associated with persistent mucosal inflammation that triggers uncontrolled immune responses. This pathology constitutes a significant public health challenge that requires novel treatments. To investigate the capacity of bioaccessible SFN in preventing or modulating the severity of IBD, intestinal inflammation was reproduced in vitro by treating a Caco‐2 monolayer model of intestinal epithelium with 25 ng IL‐1β/mL that reproduces alterations in intestinal homeostasis and activates the immunoinflammatory cascade featuring IBD^40^, including the recruitment and differentiation of immune cells into the inflammasome [40] and the secretion of a range of interleukins by the intestinal epithelium [41, 42].

To evaluate the anti‐inflammatory and immunomodulatory potential of SFN, Caco‐2 culture media were supplemented with the bioaccessible fraction of broccoli stalks (5.92 μg/g dw, equivalent to 0.100 μg/mL) at the ratio 1:10 (v/v) to achieve the final concentration of SFN in the well of 0.010 μg/mL, seeking to provide physiologically relevant in vitro data under conditions that better reflect real physiological exposure, allowing a more accurate comparison with in vivo findings [43].

#### 3.2.1 Modulation of Pro‐Inflammatory Interleukin Profile by Caco‐2 Cells

**FIGURE 1:**
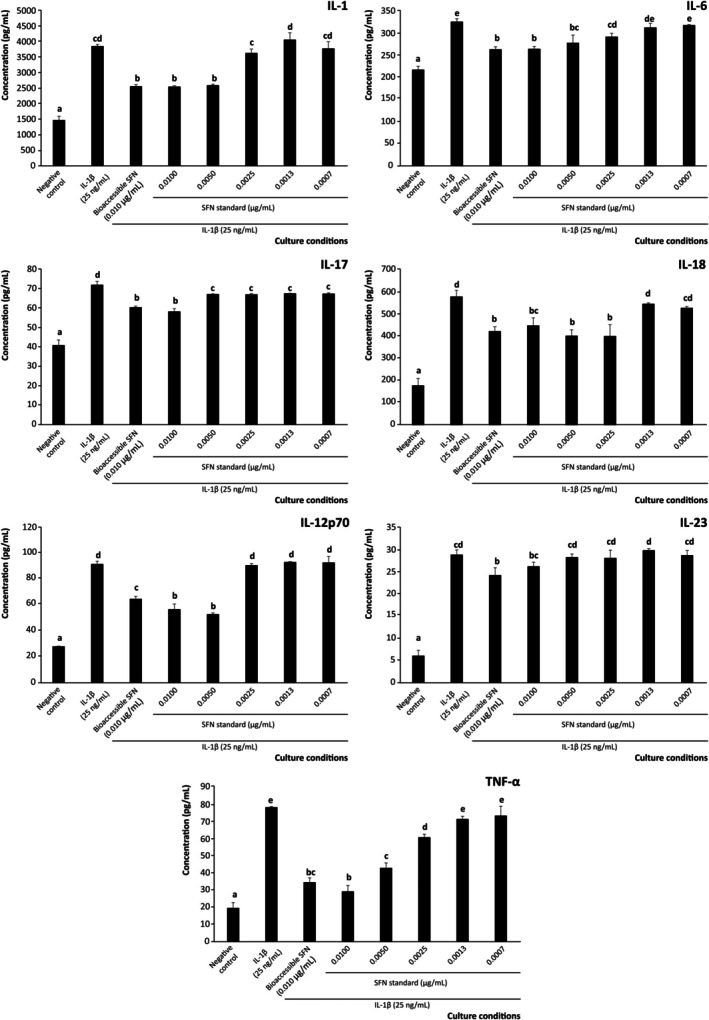
Effect of bioaccessible sulforaphane (SFN) and decreasing concentrations of pure SFN on the modulation of pro‐inflammatory interleukins (IL)‐1, IL‐6, IL‐17, IL‐18, IL‐12p70, IL‐23, and TNF‐α secretion by intestinal epithelium under inflammatory conditions. Distinct lowercase letters above each bar indicate significant differences (*p* < 0.05) according to one‐way analysis of variance (ANOVA) followed by Tukey's multiple range test (*n* = 3).

The development of the referred in vitro model of intestinal inflammation allowed recording quantifiable concentrations of IL‐1, IL‐6, IL‐17, IL‐18, IL‐12p70, IL‐23, and TNF‐*α*. Interestingly, pre‐treatment of cells with SFN at 0.0100 μg/mL (equivalent to the bioaccessible concentration) reduced the release of pro‐inflammatory interleukins by 16.0%–55.8% compared with cells exposed solely to the inflammatory stimulus (25 ng/mL of IL‐1β). The decrease observed was significant for all interleukins monitored at *p* < 0.001 (Figure 1), featuring key signaling involved in activating the autoreactive inflammation's immune settings. This capacity further highlights the relevance of brassica powder as a dietary source of anti‐inflammatory SFN, which is effective at bioaccessible concentrations in modulating the interleukin profile and thus suppressing excessive inflammatory responses in the intestinal mucosa, a key pathogenic component of IBD [44].

Despite the interest of these findings, it should be noted that when evaluating the complete bioaccessible fraction, the observed effects (attributed to SFN as the most abundant compound) may be adjusted by the presence of additional bioactive compounds released during digestion [10]. To address this limitation and retrieve evidence supporting the critical role of SFN for the anti‐inflammatory and immunomodulatory goals pursued, a model system was established using authentic SFN at a range of concentrations, including the intestinal level (0.100 μg/mL), enabling the dissection of the specific modulation of pro‐inflammatory interleukin secretion within the context of the complete digestion product [26]. The serial dilutions obtained from the referred starting concentration were further applied to supplement the culture media 1:10 (v/v) following the procedure described for the bioaccessible fraction, thus obtaining the following final concentrations in the well: 0.0100, 0.0050, 0.0025, 0.0013, and 0.0007 μg/mL.

The assessment of isolated SFN demonstrated a significant ability to reduce the secretion of pro‐inflammatory interleukins by Caco‐2 cells to a matching efficiency compared with the complete bioaccessible fraction (*p* < 0.001), thereby confirming the efficacy of bioaccessible SFN for this function (Figure 1). Notably, the bioaccessible‐like concentration of pure SFN (0.0100 μg/mL) reproduced the effects observed when cells were treated with the bioaccessible SFN. Moreover, in some cases, SFN alone was even more effective, for instance, in reducing IL‐12p70 secretion than the bioaccessible complex mixture constituting the bioaccessible fraction (*p* < 0.05, Figure 1). Starting from the bioaccessible level, testing the four additional decreasing concentrations evidenced that the operative concentrations of SFN in lowering the secretion of pro‐inflammatory interleukins were 0.0100 and 0.0050 μg/mL for IL‐1 and IL‐12p70 (both at *p* < 0.001), and up to 0.0025 μg/mL for IL‐6 (*p* < 0.01), IL‐18 (*p* < 0.001), and TNF‐*α* (*p* < 0.001) (Figure 1). Based on these results, our model demonstrated the value of SFN at bioaccessible concentrations, enabling its direct effects on the quantitative profile of inflammatory interleukins secreted by Caco‐2 cells and thus providing clearer mechanistic insight for designing nutritional or pharmacological interventions. Such delineation of compound‐specific functionality is essential for the rational design of functional foods, nutraceuticals, and therapeutics targeting intestinal inflammatory diseases, while also helping to unravel specific molecular mechanisms of action. Moreover, it contributes to defining actual “bioactive” thresholds, which are highly valuable for establishing appropriate dietary and supplement dosages, as well as for evaluating associated risks and tolerances [45].

When analyzing the meaning of the modified interleukin profile caused by bioaccessible SFN, it was noticed that the intervention tackled distinct mechanisms. Among the separate interleukins modulated, those driving general inflammation, such as IL‐1, IL‐6, IL‐12p70, and TNF‐*α*, were significantly reduced. In this regard, the ability of SFN to significantly reduce IL‐6 secretion is particularly relevant given its role as an initiator of inflammatory cascades and immune cell recruitment [46], as well as its impact on IgA‐mediated mucosal responses [47], which are closely linked to the onset of intestinal wall inflammation [48].

The TNF‐*α* is involved in the migration of immunocompetent cells responsible for autoreactive events into the inflammasome, and reducing its secretion would be effective for attenuating inflammation [44]. In addition, this result informs on the SFN capacity to maintain mucin homeostasis and prevent autoreactive immune response associated with intestinal disbiosis [49].

Notably, SFN at 0.010 μg/mL (final concentration) also suppressed the secretion of IL‐1 and IL‐12p70 (by 33.5% and 29.3%, respectively) (Figure 1). The interest in reducing the expression of IL‐1 is related to its role as an essential mediator of autoimmune and inflammatory disorders [50], characterizing the pathogenic mechanisms of IBD upon polarizing the immune response towards Th17 cell differentiation [51, 52]. Alternatively, the modulation of the IL‐12p70 level in the inflammasome is a critical immunoregulatory event that entails a fine‐tuned secretion of IFN‐*γ* and forces the differentiation of CD4^+^ T cells into Th1 cells, associated with the prevention of IBD [53]. Beyond this central function, IL‐12p70 shares immunoinflammatory mediation with another family member, IL‐23, whose secretion was also attenuated by bioaccessible SFN (although to a limited extent). This result helps to understand the contribution of SFN to maintaining a balance between immune tolerance and reactivity [54]. Indeed, its production drives the immune response towards the production of Th1‐ and Th17‐associated interleukins and thus, it has been identified as an important therapeutic target to reduce the severity of IBD [55].

Alternatively, another interleukin involved in the immune protection of the intestinal mucosa, IL‐18, was strongly regulated by SFN. The selective reduction of IL‐18, along with a more modest decrease in IL‐23 and IL‐17 secretion, suggests that SFN acts as a modulator rather than a broad suppressor of intestinal immunity responses. Given the role of IL‐18 as a key driver of IFN‐γ production and Th1 responses, its controlled downregulation may help limit excessive inflammation without compromising epithelial defence [56]. This selectivity may be advantageous, as it allows SFN to act primarily as a modulator of active inflammation rather than as a broad regulator of adaptive immunity in epithelial models, thereby attenuating inflammatory cascades without disrupting immune tolerance mechanisms in the intestinal epithelium [54].

#### 3.2.2 Modulation of Anti‐Inflammatory Interleukin Profile by Caco‐2 Cells

**FIGURE 2:**
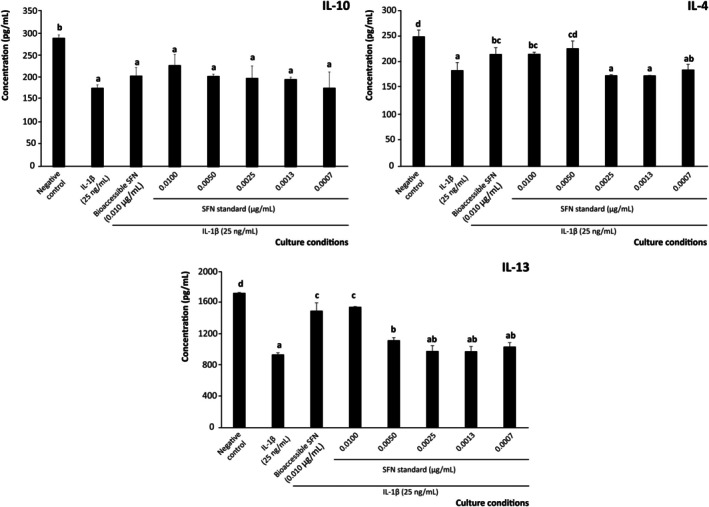
Effect of bioaccessible sulforaphane (SFN) and decreasing concentrations of pure SFN on the modulation of anti‐inflammatory interleukins (IL)‐10, IL‐4, and IL‐13 secretion by intestinal epithelium under inflammatory conditions. Distinct lowercase letters above each bar indicate significant differences (*p* < 0.05) according to one‐way analysis of variance (ANOVA) followed by Tukey's multiple range test (*n* = 3).

Beyond the secretion of pro‐inflammatory interleukins that activate the adaptive immune response in the intestinal epithelium during inflammation, evaluating the secretion of anti‐inflammatory mediators is crucial to fully elucidate the immunomodulatory capacity of SFN (Figure 2).

The inflammation induced by IL‐1β (25 ng/mL, positive control) significantly decreased the levels of IL‐10, IL‐4, and IL‐13 by 38.7%, 25.9%, and 45.7%, respectively, compared to basal conditions (negative control) (Figure 2). This result was consistent with the observed anti‐inflammatory response, reflecting the ability of SFN to suppress the overproduction of pro‐inflammatory interleukins. Hence, pretreating Caco‐2 cells with 0.010 μg/mL of SFN reduced the IL‐1β‐induced loss of anti‐inflammatory IL‐10, IL‐4, and IL‐13 by 24.3% (*p* < 0.01), 52.1% (*p* < 0.01), and 71.1% (*p* < 0.01), respectively, in cells exposed to an inflammatory environment, although this recoveries were only significant for IL‐4 (*p* < 0.05) and IL‐13 (*p* < 0.001) (Figure 2).

In the model system, treatment with authentic SFN revealed that concentrations ≥ 0.0050 μg/mL (final concentration) restored the secretion of the anti‐inflammatory interleukins IL‐4 and IL‐13 by up to 77.2% (Figure 2). This finding noted the central immunomodulatory role of SFN at bioaccessible concentrations, among the plethora of bioactive compounds (e.g., phenolics) released during gastrointestinal digestion of broccoli by‐products [57] and fully agrees with previous reports describing the ability of SFN in enhancing tolerogenic interleukins using preclinical models [58]. The relevance of this result relies on the linkage of these signaling molecules (especially IL‐4 and IL‐13) with the inhibition of macrophage migration into the inflammasome and their polarization towards anti‐inflammatory phenotypes (M2) through the activation of STAT1, STAT3, and STAT6 signaling [59]. Beyond this, the main results on SFN's ability to modulate the interleukin profile engage the adjustment of the immunocompetent recruitment and differentiation. In this connection, the significant enhancement of Th2 interleukin concentration [60] drives the differentiation of macrophages into specific subtypes designated as M2_a_ (dependent on IL‐4 and IL‐13) and M2_c_ (dependent on IL‐10). Both phenotypes could act in a coordinated manner to promote matrix synthesis, although this pathway would require the co‐administration of anti‐fibrotic compounds to achieve effective intestinal wound resolution [61].

Given the precise modulation of the inflammatory mediators by SFN released during gastrointestinal digestion and its link to macrophage‐mediated adaptive immune responses involved in IBD, the effect of this modified environment on macrophage chemotaxis and differentiation was evaluated.

### 3.3 Modulation of Macrophage Migration Into the Inflammasome by Bioaccessible Sulforaphane

Unraveling the regulatory functions of SFN on migration and differentiation of macrophages into immunocompetent cells will offer opportunities for adjusting dietary interventions to specific immune settings, depending on the pathological mechanisms identified [62]. To this end, growth media from Caco‐2 cells pretreated with the bioaccessible fraction of broccoli stalks or pure SFN, under inflammatory conditions, were assessed for the ability to reduce THP‐1 cell migration.

**FIGURE 3:**
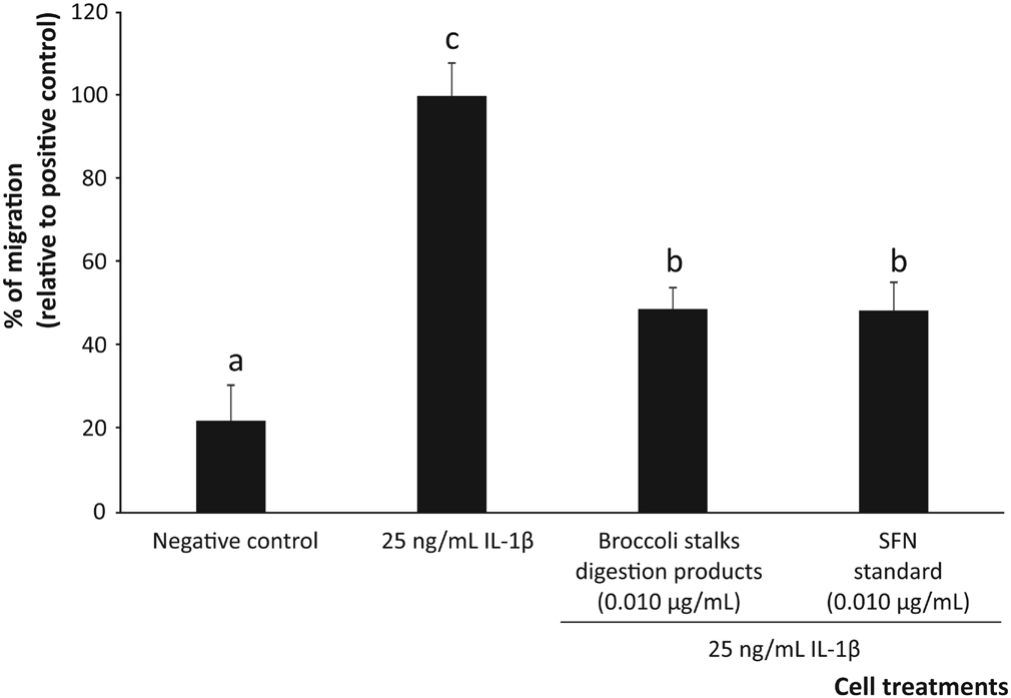
Effect of bioaccessible sulforaphane (SFN) and a matching concentration of pure SFN to modulate the percentage of macrophages induced by the interleukin cocktail produced by intestinal epithelium under inflammatory conditions. Distinct lowercase letters above each bar indicate significant differences (*p* < 0.05) according to one‐way analysis of variance (ANOVA) followed by Tukey's multiple range test (*n* = 3).

As a main finding, monocytic THP‐1 cells exposed to the supernatant of Caco‐2 treated only with the inflammatory stimulus showed a 4.5‐fold increase in migration compared to unstimulated cells. However, when treating Caco‐2 cells with SFN at 0.010 μg/mL, the migration induced by the pool of interleukins secreted was reduced significantly (*p* < 0.001) by 51.1% regardless of the inflammatory environment (Figure 3). The comparison between the bioaccessible fraction and the equivalent concentration of pure SFN at matching concentration showed no significant differences (Figure 3), indicating that the inhibitory activity observed mainly derives from SFN. In detail, when this effect was analyzed in light of the pro‐ and anti‐inflammatory interleukin profile acting as chemoattractants, specific relationships were identified according to the functionality described for the different molecules, especially regarding IL‐1β and IL‐18, which are key drivers of the macrophage recruitment and also IL‐6, IL‐17, and IL‐23, which amplify the process by promoting survival, chemotaxis and pro‐inflammatory polarization [63, 64, 65].

Among the interleukins involved in initiating the migration cascade (IL‐1 and IL‐18), the former has been associated with the expression of the macrophage migration inhibitory factor (MIF) [66]. This is a chemotactic molecule ubiquitously expressed by various cells, including intestinal epithelium, to recruit immune cells [66, 67]. Moreover, IL‐18, whose increased levels have been observed in IBD mucosal samples, is also critically involved in macrophage migration into the inflammasome. The immunological neutralization of IL‐18 by 0.010 μg/mL of SFN (final concentration) may alleviate intestinal inflammation by preventing IL‐18‐mediated disruption of the epithelial monolayer [68]. This mechanism of action of SFN would be reinforced by its capacity to inhibit the secretion of IL‐1 and TNF‐*α*, which are known to induce tight junction disruption via NF‐κB‐dependent regulation of MLCK expression [68].

In addition to the primary role in triggering macrophage migration, these interleukins may also contribute to additional aspects of the overall chemotactic response [48]. Given the reduction of IL‐6 described, the SFN treatment would lower its reactivity with transmembrane proteins (e.g., glycoprotein 130), thus tackling this route for macrophage recruitment [69]. Beyond this, the SFN's ability to inhibit the secretion of IL‐6 would contribute to controlling the autoreactive immune response in the intestinal epithelium by reducing macrophage infiltration [47]. Moreover, IL‐17 functions as a chemoattractant for macrophages [70, 71] and its neutralization could further contribute to reducing macrophage migration under inflammatory conditions [68].

### 3.4 Modulation of Macrophage Polarization Towards M1/M2 Phenotypes by Sulforaphane

The course of inflammation is closely associated with the macrophage phenotype, pro‐inflammatory (M1, CD86^+^) or anti‐inflammatory (M2, CD206^+^). After assessing the ability of bioaccessible SFN to influence the interleukin profile of intestinal epithelium and the subsequent recruitment of macrophages, its effect on macrophage differentiation was evaluated. The achievement of this objective provided evidence on the regulation of macrophage immune functions and their relevance in IBD.

#### 3.4.1 Macrophage Polarization Towards Pro‐Inflammatory (M1) Cells

**FIGURE 4:**
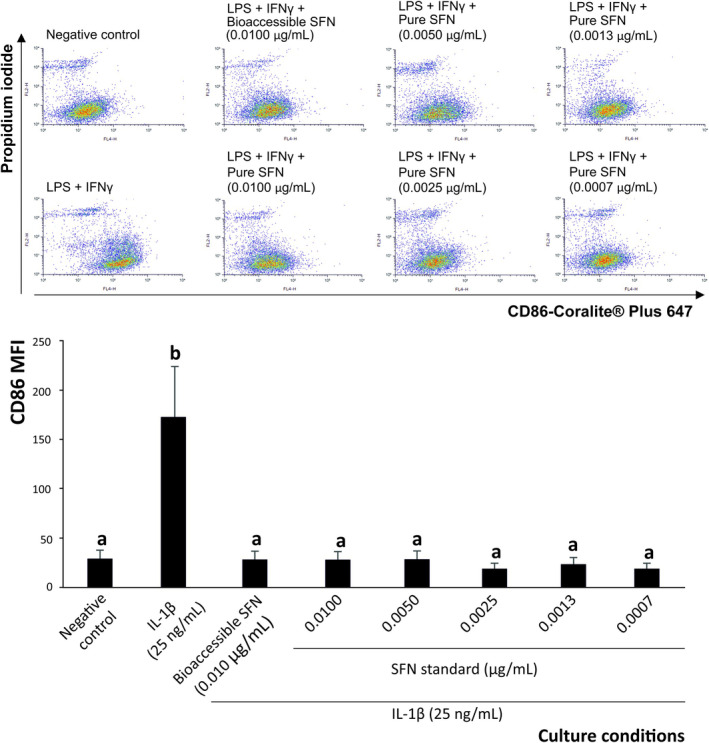
Dot plots of representative pro‐inflammatory (CD86‐Coralite Plus 647, M1) of M0 macrophages exposed to pro‐inflammatory growth media of Caco‐2 cells in the presence/absence of bioaccessible sulforaphane and decreasing pure SFN. Distinct lowercase letters above each bar indicate significant differences (*p* < 0.05) according to one‐way analysis of variance (ANOVA) followed by Tukey's multiple range test (*n* = 3).

To evaluate the ability of dietary SFN from broccoli by‐products to induce the anti‐inflammatory differentiation of THP‐1‐derived macrophages, flow‐cytometry analysis of the cell surface expression of the M1 marker CD86 (indicative of pro‐inflammatory differentiation [36]) was performed (Figure 4). Macrophages exposed to the interleukin microenvironment generated by epithelial cells after IL‐1β stimulation increased by 5.7‐fold the expression of CD86 (M1 surface marker). Interestingly, when exposing M0 macrophages to the growth media obtained after pre‐treating Caco‐2 cells with bioaccessible SNF (0.010 μg/mL, final concentration) or a solution of the pure SFN at a matching concentration and IL‐1β, this ITC reduced significantly the CD86 expression recorded when treating cells with supernatants Caco‐2 exposed to IL‐1β (*p* < 0.001) to the level observed in untreated cells that was maintained in all dilutions tested in the model system (0.0100–0.0007 μg/mL) (Figure 4).

**FIGURE 5:**
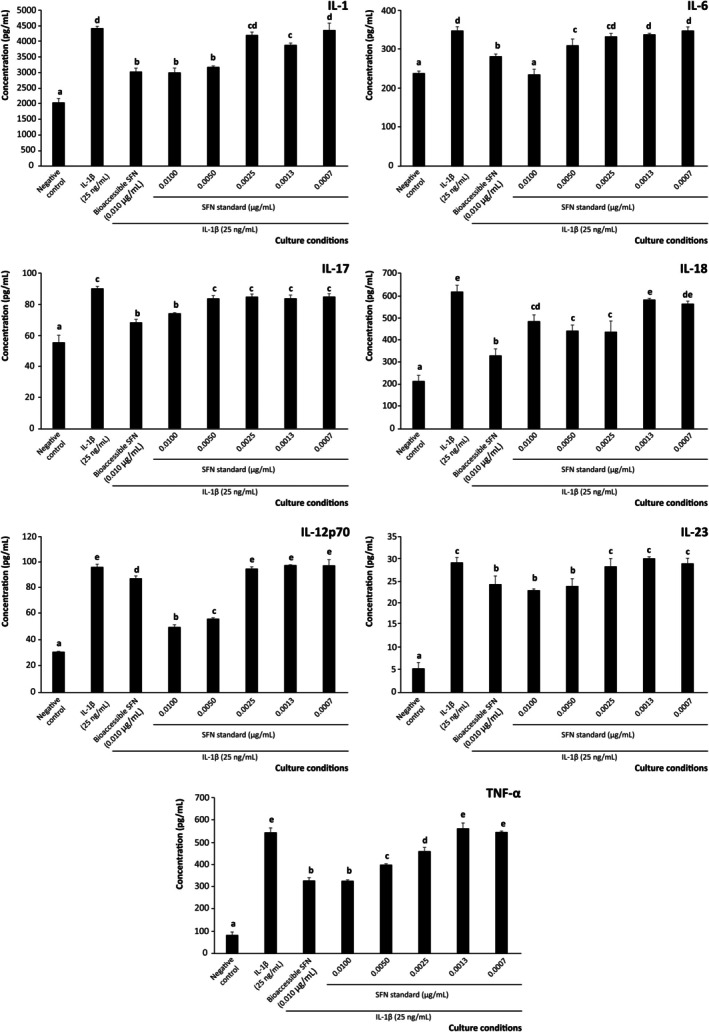
Effect of bioaccessible sulforaphane and decreasing concentrations of pure SFN on the modulation of pro‐inflammatory interleukin (IL)‐1, IL‐6, IL‐17, IL‐18, IL‐12p70, IL‐23, and TNF‐α secretion by THP‐1‐derived macrophages exposed to 25 ng/mL IL‐1*β*. Distinct lowercase letters above each bar indicate significant differences (*p* < 0.05) according to one‐way analysis of variance (ANOVA) followed by Tukey's multiple range test (*n* = 3).

In addition to the modulation of the macrophages' costimulatory phenotype, the ability of SFN to weaken the secretion of pro‐inflammatory interleukins by macrophages was determined by monitoring IL‐1, IL‐6, IL‐12p70, IL‐17, IL‐18, IL‐23, and TNF‐*α* (Figure 5), which are associated with the activation of effector cells responsible for epithelial damage and chronic inflammation in the intestine when dysregulated [72].

When profiling quantitatively the pro‐inflammatory interleukins secreted by M1 macrophages in the described environment, it was verified that SFN at 0.010 μg/mL significantly decreased the concentration of all the ILs monitored by 26.7%, on average (Figure 5). Thus, bioaccessible SFN reduced the concentration of specific pro‐inflammatory interleukins in the following decreasing order: IL‐18 (46.6%, *p* < 0.001) > TNF‐*α* (39.8%, *p* < 0.001) > IL‐1 (31.6%, *p* < 0.001) > IL‐17 (24.3%, *p* < 0.001) > IL‐6 and IL‐23 (17.9%, *p* < 0.01, on average) > IL‐12p70 (9.0%, *p* < 0.05). Nonetheless, despite this restraining capacity, no interleukin returned to the baseline values recorded in untreated samples (Figure 5).

These results show that the physiological concentration of SFN released in the intestinal lumen after digestion of broccoli by‐products has a critical capacity to modulate macrophage differentiation, thereby contributing efficiently to the modulation of intestinal barrier damage in IBD [19, 72, 73]. This new evidence complements previous reports on the anti‐inflammatory activity of SFN at supraphysiological concentrations [20, 21, 22, 23], providing a more physiologically relevant perspective. In this context, monitoring M1‐related interleukins in tissue treated with bioaccessible SFN provides insights into its potential to modulate the IBD progression. Thus, it was hypothesized that, alongside the evolving macrophage phenotype regarding the expression of surface costimulatory markers, and considering the plasticity of macrophages [58], SFN could also induce the differentiation of functional phenotypes characterized by an efficient suppression of the interleukin profile aligned with the prevention of inflammation. This ability could be linked to the previously reported capacity of SFN to enhance glycolytic and respiratory activity in macrophages, thereby reinforcing the metabolic reprogramming towards an anti‐inflammatory phenotype [23].

The ability of bioaccessible SFN within complex mixtures to inhibit the secretion of inflammatory interleukins by M1‐CD86^+^ macrophages was comparable to that of equivalent concentrations of pure SFN for IL‐1, IL‐17, IL‐23, and TNF‐*α*, whereas the pure compound was more effective for IL‐6 and IL‐12p70, achieving significant differences at *p* < 0.001 for both of them (Figure 5). This effect was maintained for selected interleukins at the final SFN concentrations up to 0.0050 μg/mL (IL‐1, IL‐6, IL‐12p70, and IL‐23) and 0.025 μg/mL (IL‐18 and TNF‐α) (Figure 5), providing valuable insight into the specific immunomodulatory pathways modulated by this bioactive ITC.

#### 3.4.2 Macrophage Polarization Towards Anti‐Inflammatory (M2) Cells

**FIGURE 6:**
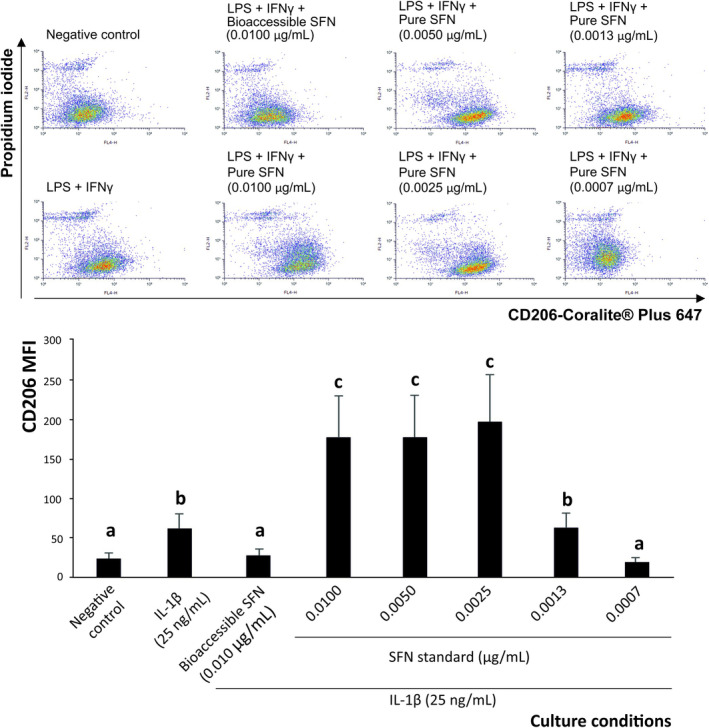
Dot plots of representative pro‐inflammatory (CD206‐Coralite Plus 647, M1) of M0 macrophages exposed to pro‐inflammatory growth media of Caco‐2 cells in the presence/absence of bioaccessible sulforaphane (SFN) and decreasing pure SFN. Distinct lowercase letters above each bar indicate significant differences (*p* < 0.05) according to one‐way analysis of variance (ANOVA) followed by Tukey's multiple range test (*n* = 3).

The immunomodulatory potential of SFN available at the intestinal lumen was evaluated through its capacity to induce an anti‐inflammatory (M2) macrophage phenotype, supporting new valorisation strategies for broccoli by‐products as a functional ingredient against intestinal inflammation. With this objective, the M2 differentiation was monitored by measuring the surface expression of CD206 after exposing M0, THP‐1‐derived macrophages to the interleukin environment generated by intestinal epithelial cells (Figure 6). The pro‐inflammatory stimulus (25 ng/mL) significantly augmented CD206 expression by 2.5‐fold (*p* < 0.01), which is associated with an anti‐inflammatory phenotype [36] (Figure 6). This costimulatory phenotype agrees with previous descriptions of the coincident occurrence of both M1 and M2 macrophages during an inflammatory process, which are activated alternatively to maintain a steady ratio needed for activating or suppressing the immune response, respectively, in vivo [74].

The supernatant of Caco‐2 cells pre‐treated with broccoli digestion products, providing a bioaccessible concentration of SFN of 0.010 μg/mL (final concentration), did not increase the frequency of CD206^+^ macrophages relative to the negative control (Figure 6). However, it is important to consider that the physiological frame of immune‐mediated diseases is the imbalance between both CD86^+^ and CD206^+^ macrophage populations [74]. In this regard, when analyzing the interference of the macrophage differentiation process by SFN during inflammation and the ratio between M1 and M2 cells, it was noted that while macrophages exposed to the growth media of untreated epithelial cells, this ratio was 5.5:4.5 (M1/M2); after inflammatory stimulation, the frequency of M1 cells increased to a ratio of 7.4:2.7 (M1/M2). Interestingly, bioaccessible SFN almost restored the level recorded under control conditions (5.0:5.0 (M1/M2)) (Figure 6), thus suggesting its ability to restore the immune homeostasis [74].

To confirm the key contribution of SFN in polarizing macrophages, a range of pure SFN concentrations was assessed regarding its capacity to enhance the frequency of M2 cells. Interestingly, it was found that SFN's concentrations from 0.0100 to 0.0025 μg/mL increased significantly (by almost 6‐fold, on average, *p* < 0.001) the expression of CD206^+^. This finding further supports the association of SFN with macrophage differentiation upon the regulation of the protein kinase B (AKT pathway) [21], which regulates the increased differentiation of M2 macrophages while inhibiting the formation of the M1 phenotype [74].

The different response observed between the bioaccessible fraction and pure SFN may result from multiple factors. In this regard, in complex mixtures, such as digestion solutions, SFN may interact with other compounds, affecting its bioavailability and cellular uptake. For instance, phenolic compounds can form complexes with SFN, potentially reducing its effective concentration and bioactivity [75]. Indeed, these additional molecules may also modulate the expression and activity of enzymes involved in SFN metabolism, further impacting its biological activity. Beyond this, the matrix composition can influence the stability and release kinetics of SFN, affecting its ability to reach target cells and activate signaling pathways (e.g., ACT‐pathway) [21]. These factors underscore the importance of considering the matrix composition when evaluating phytochemicals' bioactivity, as they help to clarify the relative contribution of an individual compound.

Although SFN was the predominant and most stable ITC released from brassica foods during digestion, the bioaccessible fractions remain chemically complex and may contain additional phytochemicals at low or trace concentrations, which could potentially exert additive, synergistic, or antagonistic biological effects [10]. To address this intrinsic complexity, the present study combined the bioaccessible fraction with pure SFN at matching concentrations and beyond (up to 0.0007 μg/mL) to estimate its specific contribution to anti‐inflammatory effects.

Therefore, while the experimental evidence supports a major role for SFN in modulating inflammatory signaling at the intestinal epithelial level, the contribution of other bioaccessible bioactive constituents of the broccoli stalk cannot be ruled out entirely. Indeed, the biological response to the bioaccessible fraction reflects the combined effect of a variety of phytochemicals, in which the evidence retrieved suggests that SFN acts as a key (but not exclusive) modulator.

**FIGURE 7:**
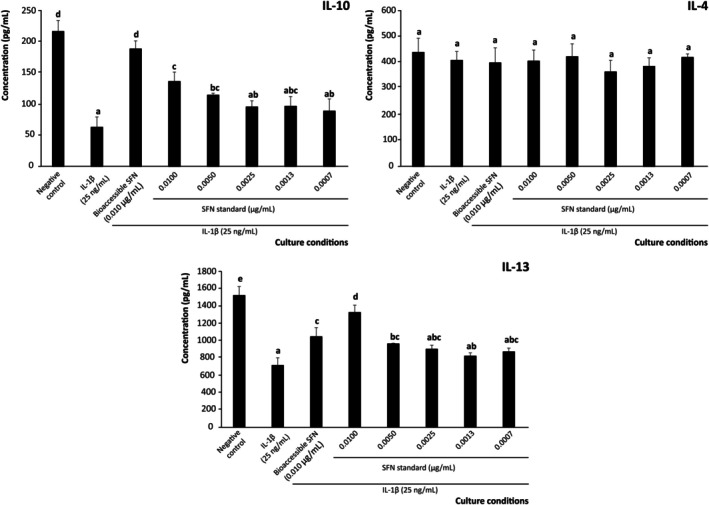
Effect of bioaccessible sulforaphane (SFN) and decreasing concentrations of pure SFN on the modulation of pro‐inflammatory interleukin (IL)‐10, IL‐4, and IL‐13 secretion by THP‐1 derived macrophages and exposed to 25 ng/mL IL‐1*β*. Distinct lowercase letters above each bar indicate significant differences (*p* < 0.05) according to one‐way analysis of variance (ANOVA) followed by Tukey's multiple range test (*n* = 3).

Concerning the quantitative profile of M2‐related interleukins, this information provides further insight into the SFN's ability to prevent inflammation during immunomediated processes. The monitored interleukins (IL‐10, IL‐4, and IL‐13) are related to tissue repair and inflammation resolution [74]. In this concern, the interleukin profile secreted by Caco‐2 cells in response to an inflammatory stimulus significantly lowered IL‐10 and IL‐13 (by almost 7.0 and 5.0‐fold, respectively, *p* < 0.001) while IL‐4 remained unchanged compared to the negative control (Figure 7). When assessing SFN at 0.0100 μg/mL (final concentration), both the bioaccessible fraction from broccoli by‐products and the pure compound prevented the decrease of anti‐inflammatory interleukins, restored nearly all IL‐10 levels and recovered IL‐13 by 41.0% with the bioaccessible fraction and 75.6% with the standard solution (Figure 7).

These findings are in line with previous studies, which reported SFN as an immunomodulator agent capable of stimulating the secretion of tolerogenic interleukins by macrophages in a context of anti‐inflammatory polarization, downregulating the JAK1/STAT1 signaling pathway [22]. However, to the best of our knowledge, the previous assessments on SFN immunomodulatory activity analyze supraphysiological concentrations (≥ 5 μM), and the complementary evidence retrieved in the present work is therefore essential to envisage the actual potential of broccoli by‐products to provide efficient SFN concentrations against IBD in vivo [76]. Hence, given the putative interleukins' functions to govern the course of inflammation, overlapping mechanisms are responsible for boosting M2 macrophage differentiation, epithelial cell restitution, and mucosal barrier reinforcement [77]. Accordingly, the main results obtained in the present work will offer new opportunities to enhance the management of immune‐mediated intestinal pathologies.

When considered together, these results extend prior evidence on SFN's anti‐inflammatory effects gathered upon in vivo and in vitro in intestinal models, represented, for instance, by its capacity to mitigate histological damage and to reduce IL‐1*β*, IL‐6, IL‐17, IL‐18, IFN‐*γ*, and TNF‐*α* or augment IL‐10 expression, and to restore microbiota/Nrf2 balance in the course of DSS‐induced colitis [12, 14, 15, 17]. However, it has to be noted that these studies used SFN doses in the micromolar range, overlooking epithelium‐macrophage crosstalk and matrix.

The comparison between the bioaccessible fraction of broccoli stalks and pure SFN revealed that physiologically relevant concentrations modulate epithelial inflammatory signaling and downstream macrophage responses. However, the differences observed between the pure compound and its concentration in the bioaccessible fraction suggest that, as previously discussed, synergistic or additive interactions with other phytochemicals released during digestion can occur. In this regard, brassica by‐products, in addition to glucosinolate‐derived metabolites, may contain a complex mixture of phenolic compounds [10], many of which exhibit independent, valuable biological activities by modulating inflammatory signaling cascades and thus influence macrophage polarization and cytokine secretion profiles [78]. Importantly, food‐matrix effects and phytochemical synergies have been shown to enhance biological responses compared with isolated compounds, particularly under gastrointestinal digestion‐mimicking conditions [26]. Therefore, although our findings indicate that bioaccessible SFN acts as a major driver of the observed immunomodulatory effects, the contribution of additional bioactive constituents within the digested broccoli matrix cannot be fully excluded.

Although our in vitro Caco‐2/THP‐1 co‐culture model has enabled mechanistic analysis of epithelial–macrophage crosstalk and the immunomodulatory effects of bioaccessible SFN at physiologically relevant concentrations, providing robust evidence, it does not fully reproduce the complexity of the human intestinal microenvironment. Traditional immortalized cell lines lack mucus‐producing cells, show barrier properties that differ from the native intestine, and do not represent the actually ongoing multicellular immune networks or microbial interactions that contribute to IBD pathogenesis. Thereby, advanced in vitro models that combine multiple cell types and immune lineages have been developed to overcome the underestimation that simple co‐cultures provide of the complex cell interaction mechanisms occurring in vivo [79]. From this perspective, the informative results obtained in the present work concerning mechanistic insights should be interpreted within the scope of controlled cellular interactions rather than definitive translational models, whose limitations should be addressed in future research.

Another important dimension in the pathogenesis of IBD and the translational relevance of SFN is associated with the gut microbiota. In this regard, a growing body of evidence has demonstrated that SFN can actively modulate microbial communities and gut metabolites in animal models, with attendant effects on inflammation and improved intestinal epithelial barrier function, thus reducing inflammation in preclinical models of metabolic disease [80]. In chemically‐induced colitis models, SFN modulated both gut microbial diversity and specific bacterial families, whereas dysbiosis is a well‐recognized hallmark of IBD pathogenesis in humans and is associated with altered inflammatory responses and epithelial dysfunction [81]. Although these microbiota‐host interactions were not directly assessed in the present in vitro work, they point to additional mechanistic layers that are likely to influence SFN's effects in vivo and merit investigation in future studies using animal, ex vivo, or organoid‐based models.

## 4 Conclusions

The present study demonstrates that bioaccessible SFN derived from broccoli by‐products exerts a dual immunomodulatory effect by reducing pro‐inflammatory interleukins and promoting anti‐inflammatory responses in an in vitro epithelial‐macrophage co‐culture. Importantly, these effects were observed at physiologically relevant concentrations, strengthening the mechanistic value of the findings and underscoring the relevance of testing bioactives at doses achievable through the diet. Although our analysis provides functional evidence that SFN can influence macrophage polarization towards an M2‐like phenotype under inflammatory conditions, the complexity of whole food matrices means that the contribution of other bioactive constituents present in the digestion products cannot be completely ruled out. However, from a translational perspective, key limitations of the study must be recognized. In this regard, it has to be highlighted that the in vitro model developed in the present work does not include a complete immune repertoire or integrate key triggers of the immune response at the intestinal level (e.g., microbiota), among other weaknesses associated with the representation of the pathogenesis and resolution mechanisms of IBD in vivo. Interactions between SFN, the gut microbiome, and host signaling pathways have been shown in animal models to modulate both microbial composition and barrier integrity, highlighting a multi‐layered mechanism that extends beyond epithelial‐macrophage crosstalk. Nonetheless, the rationale established in our research provides a robust basis for future preclinical validation.

Therefore, at the current level of evidence, future research should prioritize in vivo models of intestinal inflammation to confirm low‐dose efficacy, incorporate ex vivo human IBD tissues or organoid systems for improved physiological relevance, and explore microbiota‐host interactions as mediators of SFN's effects. Such approaches will be critical to bridge the gap between controlled cellular studies and complex organismal responses, and to inform the design of dietary intervention or therapeutic strategies targeting IBD.

## Funding

This work was supported by Ministerio de Ciencia, Innovación y Universidades (AGL2020‐120660RA‐I00, FJC2021‐046684‐I).

## Conflicts of Interest

The authors declare no conflicts of interest. Concepción Medrano‐Padial is currently enrolled in a traineeship at the European Food Safety Authority (EFSA) until October 31st, 2025. The positions and opinions presented in this article are those of the author/s alone and are not intended to represent the views or any official position or scientific works of EFSA.

## Data Availability

The data that support the findings of this study are available from the corresponding author upon reasonable request.
